# Study of Weight Quantization Associations over a Weight Range for Application in Memristor Devices

**DOI:** 10.3390/mi15101258

**Published:** 2024-10-15

**Authors:** Yerim Kim, Hee Yeon Noh, Gyogwon Koo, Hyunki Lee, Sanghan Lee, Rock-Hyun Choi, Shinbuhm Lee, Myoung-Jae Lee, Hyeon-Jun Lee

**Affiliations:** 1 Division of Nanotechnology, Daegu Gyeongbuk Institute of Science and Technology (DGIST), Daegu 42988, Republic of Korea; 2 Division of Intelligent Robot, Daegu Gyeongbuk Institute of Science and Technology (DGIST), Daegu 42988, Republic of Korea; 3 Institute of Next-Generation Semiconductor Convergence Technology (INST), Daegu Gyeongbuk Institute of Science and Technology (DGIST), Daegu 42988, Republic of Korea; 4 Department of Physics and Chemistry, Daegu Gyeongbuk Institute of Science and Technology (DGIST), Daegu 42988, Republic of Korea

**Keywords:** memristor, neural network, weight quantization, recognition

## Abstract

The development of hardware-based cognitive computing systems critically hinges upon the integration of memristor devices capable of versatile weight expression across a spectrum of resistance levels while preserving consistent electrical properties. This investigation aims to explore the practical implementation of a digit recognition system utilizing memristor devices with minimized weighting levels. Through the process of weight quantization for digits represented by 25 or 49 input signals, the study endeavors to ascertain the feasibility of digit recognition via neural network computation. The integration of memristor devices into the system architecture is poised to streamline the representation of the resistors required for weight expression, thereby facilitating the realization of neural-network-based cognitive systems. To minimize the information corruption in the system caused by weight quantization, we introduce the concept of “weight range” in this work. The weight range is the range between the maximum and minimum values of the weights in the neural network. We found that this has a direct impact on weight quantization, which reduces the number of digits represented by a weight below a certain level. This was found to help maintain the information integrity of the entire system despite the reduction in weight levels. Moreover, to validate the efficacy of the proposed methodology, quantized weights are systematically applied to an array of double-layer neural networks. This validation process involves the construction of cross-point array circuits with dimensions of 25 × 10 and 10 × 10, followed by a meticulous examination of the resultant changes in the recognition rate of randomly generated numbers through device simulations. Such endeavors contribute to advancing the understanding and practical implementation of hardware-based cognitive computing systems leveraging memristor devices and weight quantization techniques.

## 1. Introduction

Artificial intelligence finds application across diverse domains such as life sciences, communication, and transportation, rooted in the concept of mimicking brain cells, or neurons. Neural networks, the cornerstone of artificial intelligence, operate on a signaling system where stimulation passes from one cell to another [1]. The current artificial intelligence systems necessitate computerized learning and processing to address a myriad of challenges, encompassing tasks like pattern recognition and real-time processing. However, the traditional sequential processing, following the von Neumann architecture, often results in bottlenecks [2] due to the varying processing speeds of the devices. A novel approach proposes hardware [3] implementation of artificial intelligence to overcome these limitations. Recent studies comparing the efficiency of a software-based neural network [4] and a Convolutional Neural Network with hardware implementations have shown hardware to be more efficient [5]. This hardware-based artificial intelligence, mimicking human brain function, is termed neuromorphic technology [6].

The previous neuromorphic research efforts have explored innovative approaches, such as a study that devised a circuit utilizing memristors [7,8] to transfer the “weights” of a neural network [9]. Furthermore, other studies have tackled tasks like learning and recognizing a 3 × 3 alphabet by employing existing Convolutional Neural Networks (CNNs) represented as an assembly of eight-layer memristors, both in simulations and real-world implementations [10,11]. However, a notable limitation of such studies lies in the challenge of configuring the network settings in hardware-based experiments, particularly in implementing crucial factors like “weight”. In one instance, despite conducting the experiments with a simple dataset, difficulties arose in implementing the weights, leading to their conversion into binary or being represented in bit units using multiple memristors. Alternative research endeavors have explored expressing weight as variable resistance [12], although this method faces constraints in replicating the artificial intelligence performance obtained via software. Consequently, a novel technique known as “Quantization” has emerged, offering a simplified approach to handling weights [13].

Quantization involves representing the input values from a continuous range as discrete values. Another study utilized this approach to construct a model by modifying the activation function of an existing network [14,15]. For application in physical hardware, the weight levels need to be minimized, prompting the previous studies to explore binary methods utilizing memristors and circuits with variable resistors [11,12,16]. In addition, there are studies that quantized the states of LRS and HRS in order to utilize the device, and cases in which the efficiency was improved by fitting the correlation between pulse and conductance [17,18]. However, these methods suffer from reduced accuracy due to limitations in expressing numerous weights. Even in software-based implementations, binary models exhibit suboptimal accuracy [19]. To address these issues, in this work, we introduce a technique to properly quantize weights, and we propose a method to optimize them to improve accuracy. The quantization of weights is optimized by introducing the concept of “weight range”, and it is successful to reduce the values of more than three-hundred-fifty different weights to four weights. In quantization, variables are expressed in a limited number, and, at this time, how small the number of variables is that can be expressed is important. In order to control this, the concept of weight range was used, and it was found that there is a correlation between the distribution of the corresponding value and the expression value during quantization. Using this, a method to express the variables of a neural network with a small number of numbers was proposed. In addition, we discuss the system implementation of a neural network using a resistance-based memristor device.

## 2. Methods

In neural network research aimed at developing artificial intelligence, complex networks of connections between neurons are built through recurrent and regression operations on inputs and outputs. The objective is to generate an output based on a given input by leveraging numerous neurons to execute operations and transmit signals to subsequent neurons. This process involves a technique known as “backpropagation“ [1], wherein the network is trained by propagating the error of the computed value to achieve the optimal learning outcome. When performing weight updates via backpropagation, the large number of weight values that need to be integrated in individual memristor elements in an array structure makes it difficult to implement cognitive computing systems. With memristors in current stage [8,20,21,22], it is not easy to create more than 100 weight levels, and the stability of each level is unstable. Therefore, this research focuses on minimizing the number of weight levels that the trained model must represent through quantization. In this particular study, initial weights were assigned random values, and the final weights were determined through backpropagation. All computations were carried out using Python (version 3.11), with signal propagation achieved solely through addition, subtraction, and multiplication operations without utilizing any pre-defined functions. The Python version used to construct the neural network was 3.11.3, and the network was built using only the Numpy and Random packages. All operations were performed on arrays in Numpy, and sigmoid [1,23] was used as the activation function. The mean squared error method was used as the loss function in the backpropagation method [24]. This equation sets the difference between the expected value and the actual correct answer as the error and learns by correcting the square of the difference. Learning was conducted with 100 times iteration per epoch, and training was completed for 5000 epochs in total.

To validate the functionality of the computed weights on an actual device, Cadence’s electrical design automation (EDA), a semiconductor process simulator, was employed to verify the electrical signals of the physical device. To verify the actual device’s behavioral characteristics, simulations were performed using Cadence Virtuso 6.1.7. For the process design kit (PDK), we used the NSPL CMOS 0.5 μm process in the INST (Institute of Next-generation Semiconductor convergence Technology, DGIST) nano-Fab. To implement the simulation conditions, we performed mappings in the form of (weight, resistance), (input, voltage), and (output, current). For the input voltage, we added 0.65 V to account for the threshold voltage (*Vth*) of the diode used in the program. The output current was also used with the addition of a reference resistor element to keep it at a constant level. Conventional memristor devices can be used as devices for cognitive computation, and in this study, we performed PDK evaluation to implement a system by implementing resistors and diodes using Si. A quantized resistor device was fabricated and its electrical characteristics (Ketheley 2636b) and local thermal analysis (Nanoscopesystems TRM250) were measured, but only the results of PDK are shown in this study.

## 3. Results and Discussion

### 3.1. Weight Quantizaiton

To implement a system capable of cognitive computation utilizing the memristors researched to date, memristors capable of representing at least 100 or more weights are needed, and these memristors must be stable to drive and reproducible enough to distinguish the resistance between each weight level. However, the current memristors are not directly applicable to the implementation of cognitive computing systems due to the limited number of weight levels, retention problems, and endurance problems. Various problems have been raised with these memristor devices, such as the migration of oxygen atoms [25], changes in the electrical properties through the diffusion of hydrogen [26,27], the destruction of the device in localized areas by pulse signals [28], and problems with the uniformity of the memristor filaments [29]. If the number of weight levels that the model needs to represent can be minimized through quantization techniques, cognitive computing systems using memristor devices can be made easier.

**Figure 1:**
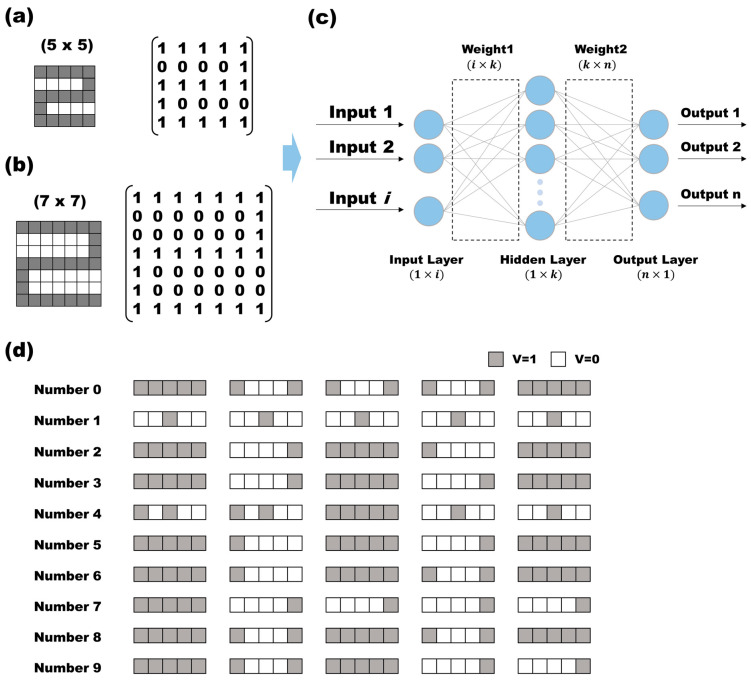
In a neural network experiment, two inputs are introduced: (**a**) a (5 × 5) array representing numbers from 0 to 9, and (**b**) a (7 × 7) array. The signal represented in the array is injected into the system in the form of a matrix. (**c**) The system used is a double neural network layer with one hidden layer. (**d**) The 10 numbers used as input were converted into 25 data.

The experimental dataset utilized both 5 × 5 and 7 × 7 numeric inputs, with a binary representation using 0 and 1. The training consisted of randomly entering ten numbers from zero to nine into a 5 × 5 or 7 × 7 grid cell. Each number was represented by a 1 in the corresponding grid cell (Figure 1a,b). In the case of the 5 × 5 input, marking ‘2’ would be depicted as ([1,1,1,1,1], [0,0,0,0,1], [1,1,1,1,1], [1,0,0,0,0,0], [1,1,1,1,1]) (Figure 1d). Subsequently, the data were flattened and fed into a single input array. The 5 × 5 input initially entered the input layer as a (1 × 25) array and underwent sigmoid computation with a weight1 of size (25 × 10) and an activation function, resulting in a hidden layer of size (1 × 10). The data then proceeded through another layer with weight2 of size (10 × 10) before undergoing sigmoid computation once more. The output layer provided a probability value ranging from zero to one for each digit, selecting the highest probability and converting it into a (1 × 10) output where one element was represented by 1. For the 7 × 7 numeric input, the input data took the form of (1 × 49), with the subsequent weights determined as (49 × 10). The succeeding hidden layers followed the same format as the 5 × 5 input, with sizes of (1 × 10) for the first layer and (10 × 10) for the second layer. Among these parameters, the values in the input layer remained fixed throughout the backpropagation process, with only weight1 and weight2 being quantized subsequently. During the quantization, weight1 and weight2 were not treated individually but instead combined. This combined set of weight1 (W1) and weight2 (W2) is denoted by W.

**Figure 2:**
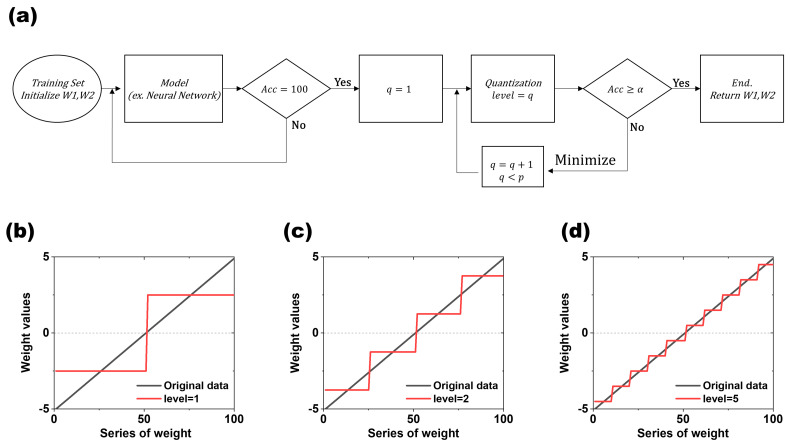
Weight quantization and process flow: (**a**) W1 and W2 fed through the training set are trained via backpropagation and move to the next step when they reach 100% recognition. They sequentially increase the quantization level (q) to find weights that satisfy α. (**b**) Conceptual diagram of weight quantization at quantization level 1, (**c**) at level 2, and (**d**) at quantization level 5.

Figure 2a illustrates the flowchart detailing the optimization and quantization process for each weight within a 5 × 5 input dataset. In the unconstrained case, achieving a 100% recognition rate for randomly input values necessitated approximately 500,000 times the backpropagation for the values of W1 and W2. We initially trained the model with 100% accuracy because the 5 × 5 and 7 × 7 data are relatively simple datasets consisting of 0s and 1s, so the accuracy can easily reach 100% if the training volume is sufficient. After sufficient training, we moved on to the next step, quantization. In this process, we only used W, which stands for W1 and W2 among the model parameters, to perform the quantization. The subsequent quantization process aimed to represent a continuum of values divided into specific steps. The key components for implementing this include the “weight range” (Equation (1)) and a “level (*q*)” for quantization. *q* is a number more than 0 and has a range below *p*, which is the maximum value.

Figure 2b–d are the result of the quantization dependent on level (*q*) (*q* = 1, 2, and 4). The black line is the original value, and the red line is the value after the quantization. As the level (q) increases, the number of steps increases, which makes the difference between the values before and after the quantization smaller. The level (*q*) serves as the target value for converting weights from their original analog form to the discrete values aimed for during quantization. The weight range represents the difference between the minimum and maximum values of W, expressed as the sum of W1 and W2 (Equation (2)). The interval is defined as the weight range divided by the desired level (*k*), indicating the range of values represented by each single level (Equation (3)). The value quantizing the actual range value at *level_(i, middle)_* is then determined by multiplying the interval by a value of *i +* 0.5 and min(*W_total_*) (Equation (4)).

(1) $$Weight\;range=\max\left(W_{total}\right)-\min\left(W_{total}\right)\;$$

(2) $$Weight\;rangeA=\max\left(\left|W_{total}\right|\right)-\min\left(\left|W_{total}\right|\right)$$

(3) interval=(Weight rangeA )/(level (q) )

(4) $$\begin{array}{c} {level}_{i,\;middle}=\min\left(W_{total}\right)\times interval\times \left(i+0.5\right)\;,\\ i=1,\;2,\;\cdots ,\;p\left(0<q<p\right)\;, \end{array}$$

where, *i* is the number of levels. Equations (1), (3), and (4) are used when the list of values being quantized consists of only positive numbers greater than zero. In this case, “Weight rangeA” in Equation (3) and “Weight range” in Equation (1) have the same value because all the numbers are positive. Conversely, if the list of values being quantized contains both positive and negative numbers, “Weight rangeA” is used to set the interval.

The initial weight values used in each experiment were used as seed values to consider 60 cases, and these values were randomly generated. The process of generating and learning a new initial weight was repeated 100 times for each individual seed value. Thus, 6000 models were created for each dataset, with each model subsequently quantized to assess the results. The quantization was performed by starting with *q* = 1 and incrementing the value of q sequentially until an optimal value of q was obtained that satisfied α (α is the target recognition rate accuracy). To proceed with the quantization while ensuring accuracy, if the recognition rate accuracy value is greater than or equal to the value of α, move to the next step; otherwise, *q* is added to 1 and the quantization is performed again. In this experiment, α was set to 100%, and, in general, the smaller α is, the lower the level value that can be reached. The quantization process runs under the condition that *q* < *p*, with the maximum value set to *p*, and the quantization process terminates when *q* exceeds *p*, as shown in the flowchart in Figure 2. Although all the points of *level_(i, middle)_* are established by *p*, there are cases where *level_(i, middle)_* is not generated in a certain interval because the number of W does not exist between those intervals. In this study, the non-generated cases were not considered when determining the final level.

**Figure 3:**
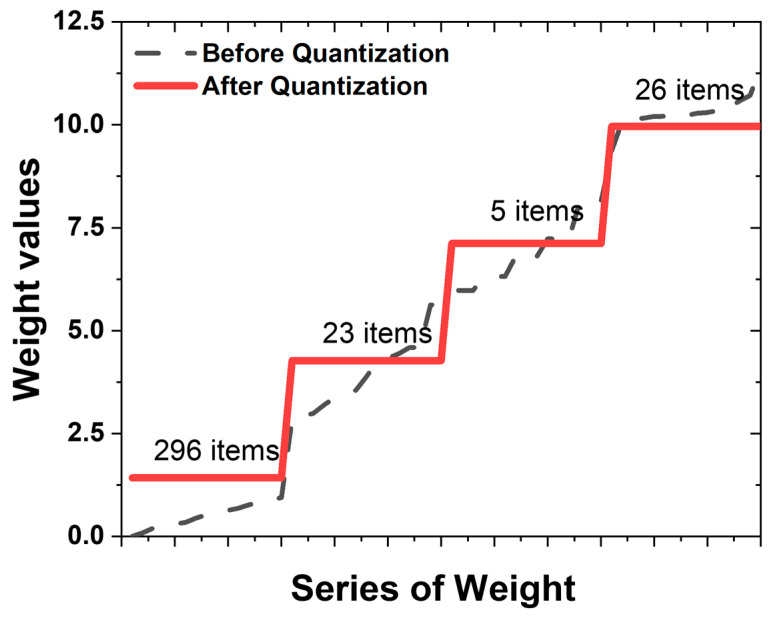
Distribution of weight values and quantization levels: (dashed line) distribution of weight values before the quantization process; (red line) quantization of 350 different weight values into 4 values.

After training the neural network based on the 5 × 5 and 7 × 7 numeric input datasets, quantization was performed based on the calculated weights. Of the parameters generated from the 5 × 5 numeric dataset, only W1 and W2 were used for the quantization. There are 350 numbers in W as an array, including 250 W1 and 100 W2. An array of W generated from a 7 × 7 number dataset has 590 numbers. The weight quantization procedure outlined in Figure 2 was implemented for the 5 × 5 dataset. Following the division of the 350 weights generated by the 5 × 5 dataset into four levels, the recognition rate remained unchanged (Figure 3). This signifies a substantial reduction of 98.9% in the weight levels compared to their original state. Similarly, for the 590 variables in the 7 × 7 dataset, a reduction of 99.3% was observed, resulting in a reduction of four levels. Figure 3 illustrates the distribution of the 350 weights derived from a single 5 × 5 dataset. The *x*-axis is arranged in ascending order of weights, while the *y*-axis represents the corresponding weight values at each point. These weights vary from 0 to 12.5, with a prevalence of lower values. The dashed black line represents the unconstrained weight distribution, exhibiting 350 distinct values. By segmenting the 350 weight values into four levels, the quantized weights, depicted in red in Figure 3, were derived. The first weight level condenses the use of 296 distinct weight values into a single value, while the second, third, and fourth levels amalgamate 30 or fewer weight values into a singular value. It is noteworthy that, while many of the values are close to zero, the levels are created not only for these values but also for those with lesser numerical significance. Specifically, the distribution containing 296 items exhibits a high distribution of values, whereas the distribution with only five items demonstrates a notably lower distribution. It is important to note that not all 6000 models employed in this experiment underwent quantization with four weights. Only in some datasets was it computationally observed to maintain a 100% recognition rate even after reducing the weights to four levels. Based on the experimental results, the stable reduction in the weight levels varied depending on the magnitude of the maximum and minimum values of the weights, which aligns with the definition of “Weight range” explained earlier.

### 3.2. Weight Range

When designing and implementing actual neural network systems in hardware, the challenge is to implement them while maintaining the accuracy of the various weights. Quantization, which is used to compensate for this drawback, has the advantage of reducing the complexity and implementation difficulty when implemented in hardware (using a memristor device). When designing a neuromorphic model, the neural network model must be finite, and, in the process, all the parameters must be represented by elements, which increases the processing difficulty of the elements, increases the complexity, and increases the cost of implementation. An algorithm used to address this is quantization, which is commonly used in the field of reducing model complexity and increasing performance.

**Figure 4:**
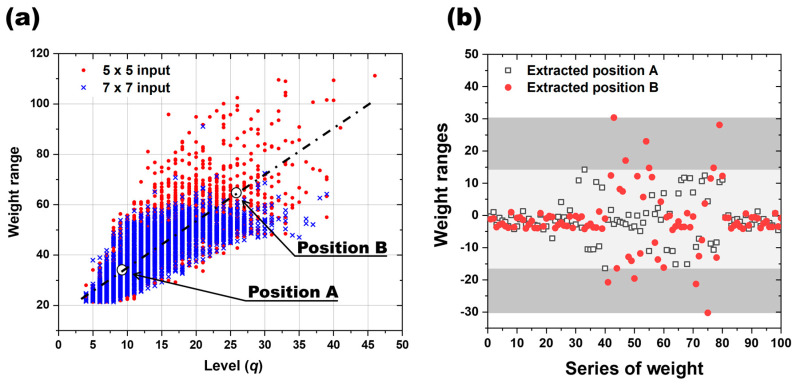
Distribution and influence of weight range: (**a**) influence of the number of quantized levels (k) on the distribution of weight range; (**b**) distribution of two different weight ranges, A and B.

The existing quantization technique offers the advantage of converting the single-precision floating point (FP32) to a half-precision floating point (FP16) or normalizing the layer [14]. This technique reduces the number precision by decreasing the calculation bits while maintaining the range, aiming to cut the memory use and computational complexity. Split into FP32 and FP16, FP32 employs 32 bits, while FP16 uses 16 bits. FP16 offers reduced memory and increased throughput in software but minimal advantages in hardware, where FP32 and FP16 provide no practical benefits. To address this, our proposed quantization technique emphasizes actual hardware device implementation, simplifying the model’s weight into a straightforward list of values. Notably, our study focuses on minimizing the unique weights by setting a low level and conducting quantization. Therefore, in order to reduce the level, the correlation was investigated using the concept of weight range mentioned above. Figure 4a presents the outcomes of an experiment aimed at determining the optimal levels for various weight ranges. The initial weights were seeded from one to sixty, and 100 iterations were conducted to generate 6000 distinct models for 5 × 5 and 7 × 7 inputs, respectively. The correlation between the weights employed in each model and the resulting number of levels in the final weights was examined. The number of levels in the final weights was defined as the point at which the recognition rate reached 100%, denoted as *q* when α is 100% in Figure 2a. Notably, the minimum level achievable for the quantized levels (*q*) appears to be directly proportional to the weight range. For both the 5 × 5 and 7 × 7 input systems, models with α satisfying 100% are found at *q* = 4, and it has been observed that weight ranges have significantly lower values of *q* around 21. An interesting aspect is the phenomenon of having the same weight range but different values of *q* level. This suggests that there are other factors that determine the level of *q* along with the weight range. For models with a weight range greater than 60, the value of *q* is determined between 10 and 40. The points A (8, 32) and B (25, 62), shown in Figure 4a, were plotted in Figure 4b for a representative model with weight ranges of 32 and 62. As already mentioned in Figure 3, the weights of the 6000 models used in this experiment are mostly clustered around zero, and this trend becomes more pronounced as the weight range increases.

**Figure 5:**
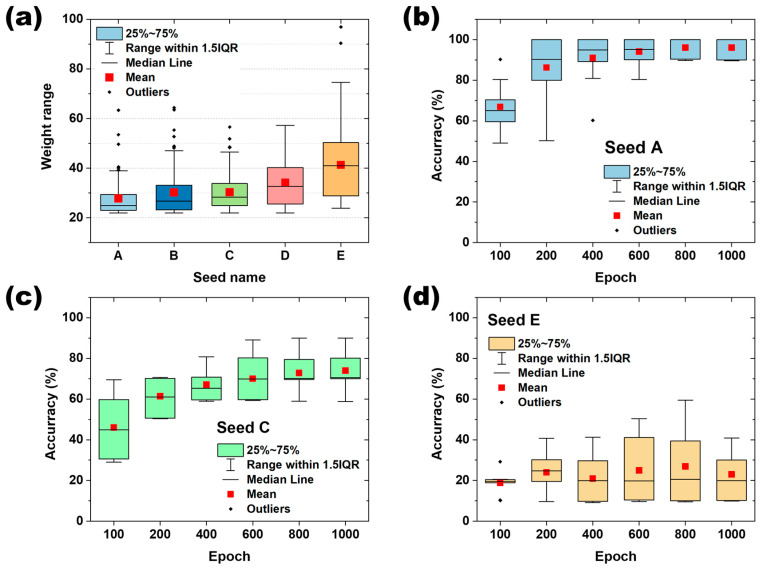
Classification of “seeds” according to weight range and the recognition rate accuracy for each seed: (**a**) configure seeds with different weight ranges in 5 zones, where the weight range is gradually increased from A to E. (**b**), (**c**), and (**d**) show the change in recognition rate as the neural network is trained on Seed A, Seed C, and Seed E, respectively.

In this comprehensive analysis encompassing 6000 models, a significant correlation between “weight range” and “*q*”, the quantizable level, was established. For the same input signal, the weight range can be an important factor in determining *q*. In addition, for the system satisfying quantization level *q* = 4, the change in the recognition rate as a function of the weight range over the number of epochs was calculated as shown in Figure 5. The relationship between quantization level (*q*) and weight range was studied by comparing the post-quantization recognition rate accuracy for 10 randomly selected models in different training states using five seed values. Figure 5a illustrates a graph depicting the distribution of the training weight ranges, where 100 models were generated for each “Seed”. The figure shows a sequential increase in both the mean value and distribution of the weight ranges from Seed A to Seed E. These seeds are integral for refining the initial weights and facilitating the subsequent training. The outcomes of computing the recognition rate accuracy based on the initial weights categorized by their respective weight ranges for Seeds A, C, and E are presented in Figure 5b–d, respectively. The accuracy of the recognition rate was evaluated as the number of computations increased for each of the three seeds in a system satisfying quantization level *q* = 4. Notably, for ‘Seed A’, characterized by the smallest weight range, the recognition rate accuracy demonstrated a consistent upward trend with each epoch, exceeding 90% accuracy after 400 epochs, as depicted in Figure 5b. ‘Seed C’ showed the same trend of gradual improvement in recognition rate accuracy as ‘Seed A’ as the number of epochs increased, with a recognition rate accuracy of about 70% after 600 epochs. After that, there was no further improvement in the recognition rate with increasing epochs, as shown in Figure 5c. In contrast to Seeds A and C, Seed E demonstrated a notable absence of improvement in the recognition rate with the advancement of training epochs. Initiating at the initial accuracy level of 20%, it maintained this accuracy level throughout the duration, even at epoch 1000, as illustrated in Figure 5d. This evaluation was performed by artificially implementing seeds with a very large range of weight ranges, which shows that the weight range quantization level, q, of the weight can have a direct impact on the recognition rate.

### 3.3. Circuit Implementation

**Figure 6:**
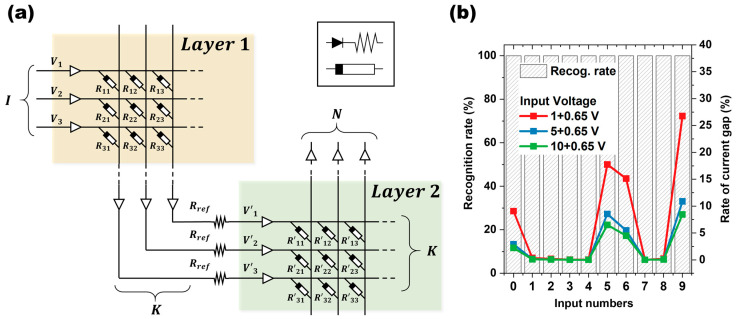
(**a**) A schematic diagram of the circuit to drive the actual device using PDK simulation; (**b**) the rate of the final current gap value from the neural network computational calculation and the number recognition rate of the device obtained from the PDK simulator.

So far, it has been shown through neural network calculations that a 100%-digit recognition rate can be obtained by using the relationship between weight range and quantization level *q*. The device simulation for an actual device chipset was completed via PDK. The PDK simulation was performed by selecting the weight that converges to the lowest *q* level of 4 in the computational calculation. The circuit is shown in Figure 6a, where the input is represented by voltage and the output is represented by current. Compared to Figure 1, *I* is mapped to *i*, *K* to *k*, and *N* to *n*. Between layers 1 and 2, a reference resistor is used to convert the current to voltage and to compensate for the current. In Figure 6, the reference resistors used for each line are all the same. To check the influence of the threshold voltage of the diode added to prevent sneak current, the difference between the recognition rate and the output current as a function of the magnitude of the input voltage was compared through circuit simulation. Figure 6b shows the accuracy of the recognition rate for the final output of the PDK simulation and the difference between the current computed by the neural network and the PDK computation. The accuracy shows the digit recognition rate for 10 different inputs (0–9), with a 100% recognition rate for all the input values. This experiment shows that even a system consisting of only four weights, which is reduced to four by quantizing the weights when building a chip that runs on a real device, can still provide reliable digit recognition. The rate of the current gap shown in Figure 6b represents the difference between the output current from the PDK simulator for device fabrication and the computational calculation for the neural network. The graph shows that the input number “9” has the rate of current gap 27% (red line in Figure 6b, using a 1 V input voltage). It can be seen that input numbers 0, 5, and 6, including 9, have more than 10% gaps regarding the output value compared to the computational neural network calculation. These gaps are the difference between the calculated output current value and the current value in the PDK simulation for driving the actual device, so they do not contribute much to the decrease in recognition rate. This trend was similar when increasing the data representing the input numbers (increasing the input information in 5 × 5 inputs). This shows that the output current value maintains a certain margin, ensuring accuracy even in different environments (gaps generated by random sources; in this case, gaps generated by device fabrication).

The difference in the current values between the neural network calculation and the PDK simulation is due to the diodes used as selectors. The diode used in this study has a threshold voltage of 0.65 V, so, if the input voltage is similar to this, the threshold voltage of the diode will be affected. As it goes through the circuits of W1 and W2, the value of the output current for each matrix slightly deviates from the calculated value, and the difference is clearly revealed in the final output current. The blue and green lines in Figure 6b show “the current gap” when the input voltage is increased to 5 V and 10 V, respectively. It can be seen that the ratio of the current gap gradually decreases as the input voltage increases. In a computer calculation, the output current is (V-Vth)/R, but, in a PDK simulation, the voltage actually changes across the resistor after it passes the diode’s threshold voltage and undergoes feedback in the circuit.

**Figure 7:**
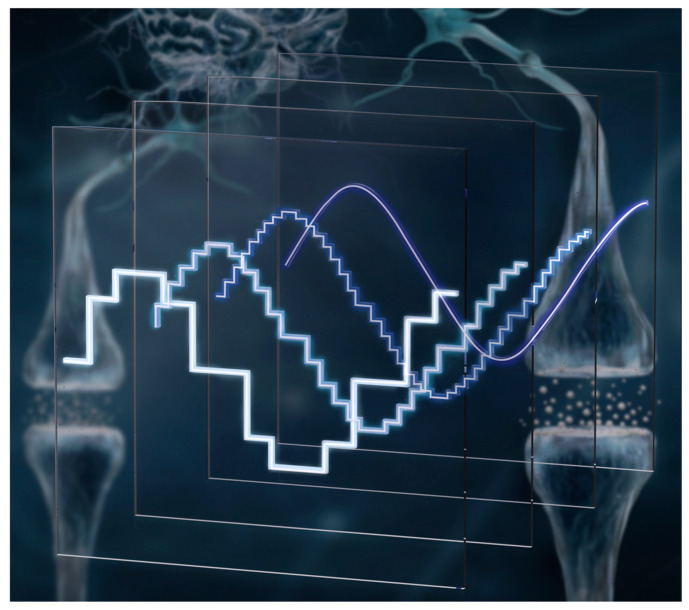
In object recognition via cognitive operations, a large amount of weights containing traditional decimal forms are simplified by quantization. Quantized weights not only reduce the amount of memory in neural network computations but can also reduce the number of weight levels that a device must represent in neural network computations implemented with memristor devices.

Quantization of weights in neural network research refers to the process of reducing the precision of the weights in a neural network. In neural networks, weights are typically represented as floating-point numbers, which require a certain amount of memory and computational resources to store and process. In general, quantization aims to reduce the memory and computational requirements of a neural network by representing the weights with fewer bits. The application of quantization to simple inputs (a 5 × 5 or 7 × 7 matrix) in this study is aimed at reducing the computational requirements of the neural network’s memory and reducing the number of weights that need to be implemented by memristors as much as possible in the application of hardware-based systems, thus facilitating the implementation of device-based neural network systems (Figure 7). Quantizing weights is essential for deploying neural networks on resource-constrained devices such as mobile phones, IoT devices, and embedded systems. However, quantization can lead to model inaccuracy, which requires careful optimization and tuning to mitigate. To quantize the weights, we introduced the concept of weight range, which provides the possibility to adjust the number of levels that can be quantized. We evaluated the cognitive operation for relatively simple digit recognition using 5 × 5 (25 inputs) and 7 × 7 (49 inputs) inputs and found a 100% recognition rate, but further evaluation of the applicability of this system to large input data is needed. Also, quantization can lead to model inaccuracies, which require careful optimization and tuning to mitigate.

## 4. Conclusions

We investigated weight quantization within the context of neural network computation and device simulation. Weight quantization was applied to compare the recognition rates in a dual-layer neural network structure using simple numeric input signals consisting of 5 × 5 and 7 × 7 matrices. In particular, we introduced the concept of weight ranges to optimize the quantization efficiency, demonstrating its correlation with weight quantization and the ensuing recognition rates. It was shown that, by simplifying the weights while maintaining the accuracy above a certain value set during quantization, the number of numbers that need to be represented by weights can be reduced by more than 95% while maintaining a certain level of recognition rate. The device simulations were performed by applying the quantized weights to an array of two neural network layers, and it was shown that the number recognition rate can be stably secured even in actual device operation.

Overall, the efforts to implement neural network computation with memristor devices are still ongoing. However, the implementation of memristor-based neural network computation systems utilizing the devices currently being researched is not easy due to the instability of the devices, high stability, and the requirement for functionality under many conditions. Advancing the quantization technology presented in this study is expected to enable the implementation of artificial intelligence systems with high energy efficiency. In particular, a prototype memristor-based cognitive computing system for simple systems will be realized through further research.

## Data Availability

The data presented in this study are available upon request from the corresponding author.
